# Predictors for Unsuccessful Reductions in Hemodialysis Frequency during the Pandemic

**DOI:** 10.3390/jcm12072550

**Published:** 2023-03-28

**Authors:** Suthiya Anumas, Sithichai Kunawathanakul, Pichaya Tantiyavarong, Pajaree Krisanapan, Pattharawin Pattharanitima

**Affiliations:** 1 Chulabhorn International College of Medicine, Thammasat University, Pathum Thani 12120, Thailand; 2 Division of Nephrology, Department of Medicine, Faculty of Medicine, Thammasat University, Pathum Thani 12120, Thailand; 3 Department of Clinical Epidemiology, Faculty of Medicine, Thammasat University, Pathum Thani 12120, Thailand

**Keywords:** COVID-19, hemodialysis, reduction dialysis frequency, prediction

## Abstract

Background and Objectives: Patients receiving in-center hemodialysis are at a high risk of coronavirus disease 2019 (COVID-19) infection. A reduction in hemodialysis frequency is one of the proposed measures for preventing COVID-19 infection. However, the predictors for determining an unsuccessful reduction in hemodialysis frequency are still lacking. Materials and Methods: This retrospective observational study enrolled patients who were receiving long-term thrice-weekly hemodialysis at the Thammasat University Hospital in 2021 and who decreased their dialysis frequency to twice weekly during the COVID-19 outbreak. The outcomes were to determine the predictors and a prediction model of unsuccessful reduction in dialysis frequency at 4 weeks. Bootstrapping was performed for the purposes of internal validation. Results: Of the 161 patients, 83 patients achieved a dialysis frequency reduction. Further, 33% and 82% of the patients failed to reduce their dialysis frequency at 4 and 8 weeks, respectively. The predictors for unsuccessful reduction were diabetes, congestive heart failure (CHF), pre-dialysis overhydration, set dry weight (DW), DW from bioelectrical impedance analysis, and the mean pre- and post-dialysis body weight. The final model including these predictors demonstrated an AUROC of 0.763 (95% CI 0.654–0.866) for the prediction of an unsuccessful reduction. Conclusions: The prediction score involving diabetes, CHF, pre-dialysis overhydration, DW difference, and net ultrafiltration demonstrated a good performance in predicting an unsuccessful reduction in hemodialysis frequency at 4 weeks.

## 1. Introduction

Coronavirus disease 2019 (COVID-19) was declared a pandemic on 11 March 2020 [1] by the World Health Organization (WHO), and it has affected millions worldwide. The mortality-related risk factors of COVID-19 are chronic comorbidities, including diabetes, hypertension, chronic obstructive pulmonary disease (COPD), cardiovascular disease, obesity, cancer, and chronic kidney disease, especially in those who were suffering from end-stage kidney disease (ESKD) and receiving dialysis treatment [2,3,4].

COVID-19 spreads via droplet transmission from coughing, sneezing, speaking, and breathing [5]. It also spreads via direct contact with the eyes, nose, and mouth, or through the air over a short range (short-range airborne transmission). However, in a crowded indoor setting where people spend time for extended periods and/or in a poorly ventilated environment, infectious particles remain in the air for a longer duration of time and travel farther than usual, thus resulting in long-range airborne transmission [5]. A dialysis unit is compatible with the aforementioned setting, which thus results in a higher risk of COVID-19 infection in hemodialysis patients.

Reducing hemodialysis frequency might diminish COVID-19 exposure in either patients or dialysis staff, reduce dialysis staff work, increase the space between patients, reduce the amount of public transportation used, and conserve personal protective equipment (PPE) [6]. Although a reduced long-term hemodialysis frequency may result in an inadequate dialysis, especially in patients with a residual renal urea clearance of less than 2 mL/min/1.73 m^2^ [7], a reduced short-term hemodialysis frequency during a pandemic might be beneficial. Some guidance has suggested consideration of a reduction in hemodialysis frequency from thrice to twice weekly in patients who are able to tolerate this reduction as one of measures for managing hemodialysis patients during the COVID-19 pandemic [8]. However, there has been limited evidence by which to determine the effect of reducing short-term dialysis frequency and the predictors for an unsuccessful reduction in dialysis frequency during the pandemic. Thus, we conducted this study to determine the predictors of unsuccessful reduction and to develop a clinical prediction score in order to determine the risk of unsuccessfully reducing dialysis frequency in a pandemic setting.

## 2. Materials and Methods

This retrospective observational study utilized data from the dialysis unit at the Thammasat University Hospital, Thailand. Ethical approval was granted by The Human Research Ethics Committee of Thammasat University: Medicine (111/2565). All adult ESKD patients who were receiving thrice-weekly hemodialysis in 2021 for at least one week before a decrease in dialysis frequency to twice-weekly hemodialysis were included in the study. The exclusion criteria were patients who (1) received the first hemodialysis session after 5 July 2021, which was the date of starting a reduction in dialysis frequency; (2) received their last hemodialysis session before 5 July 2021; and (3) had no dialysis data within one week prior to decreasing their dialysis frequency.

The primary outcome was determining the predictors of an unsuccessful reduction in hemodialysis frequency at 4 weeks, which was defined as a failure to maintain twice-weekly hemodialysis sessions for 4 weeks and the need to transfer back to thrice-weekly hemodialysis for any reason. The secondary outcome was to determine a prevalence of unsuccessfully reducing dialysis frequency at 4 and 8 weeks as well as to create a clinical prediction model score for the unsuccessful reductions in dialysis frequency.

All hemodialysis patients who met the eligibility criteria were identified from an electronic hemodialysis database. We retrieved demographics, laboratory data, and dialysis parameter records. The baseline demographic variables, including age, sex, vascular access, and comorbidities, were retrieved. The latest laboratory data within 90 days prior to dialysis frequency reduction, including hemoglobin, electrolytes, calcium, phosphate, parathyroid hormones, albumin, dialysis adequacy parameters, and last dry weight (as measured by bioelectrical impedance (BIA) within 90 days before decreasing frequency), were included. Pre-dialysis overhydration was defined as the mean of pre-dialysis body weight minus the dry weight from BIA. Post-dialysis overhydration was defined as the mean of post-dialysis body weight minus the dry weight from BIA. The mean value of dialysis parameters—including net ultrafiltration, pre- and post-dialysis body weight, blood pressure, and heart rate within one week prior to decreasing dialysis frequency—were included. All patients were provided with education for fluid and protein-restricted diets; additionally, diuretics were given to those patients who had residual urine outputs. All patients were prescribed 4-h dialysis treatment times, with a dialysis prescription at the discretion of the attending nephrologists. The causes of unsuccessful reductions in dialysis frequency were reported.

### 2.1. Statistical Analysis

The categorical data were presented in frequency and percentage. The numerical data were presented in the median and interquartile range (IQR). The medians were compared using a Wilcoxon rank-sum test, whereas the proportions were compared using Fisher’s exact test. The logistic regression analysis was performed to determine the predictors for an unsuccessful reduction in dialysis frequency. Non-missing variables with a *p*-value of ≤0.1 from a univariate logistic regression analysis were included in a multivariate logistic regression analysis. The strength of association between the predictors and outcome was reported as an odds ratio (OR) and a 95% confidence interval (95% CI). A two-sided *p*-value of <0.05 was considered statistically significant. All statistical analyses were performed using the STATA version 17.0/BE.

### 2.2. Model Development 

The predictors from the multivariate logistic regression analysis were included in a developed model. The internal validation was assessed by a bootstrapping procedure [9], with a 500-bootstrap sample in order to quantify the optimism of the developed model. The model was then adjusted by a shrinkage factor to create a final model. The log odds from the final model were used to create a prediction score. The area under the receiver operating characteristics curve (AUROC) was calculated to determine the performance of the developed model, final model, and the prediction scores.

## 3. Results

### 3.1. Baseline Characteristics

Of the 161 hemodialysis patients in the dialysis unit at Thammasat University Hospital in 2021, 78 patients were excluded: 19 patients received their first hemodialysis session after 5 July 2021, 6 patients received their last hemodialysis session before 5 July 2021, 4 patients had no data within one week prior to their decreasing frequency, 18 patients received hemodialysis twice a week, and 31 patients continued hemodialysis thrice a week due to the treating physician’s decision. Of the 83 included patients, 56 patients successfully reduced their hemodialysis frequency (67%) for 4 weeks. 

The median (IQR) age of the included patients was 69.6 (63.1–80.4) years. Further, 53% of the patients were female. The most common vascular access was via the arteriovenous fistula (65.1%). Hypertension, dyslipidemia, and diabetes mellitus (DM) were found in 96.4, 66.3, and 57.8% of patients, respectively. There was significantly higher proportion of DM patients in the unsuccessful group (77.8%) than in the successful group (48.2%). In addition, a numerically higher proportion of patients with congestive heart failure (CHF) was observed in the unsuccessful group (14.8%) than in the successful group (3.6%). The median bicarbonate and intact parathyroid hormone (iPTH) level was found to be lesser in the unsuccessful group; however, the data were not available for some patients. The dialysis adequacy was not significantly different in both groups. However, pre-dialysis overhydration was significantly greater in the unsuccessful group. The dry weight from BIA, actual set dry weight, and the pre- and post-dialysis body weight were numerically higher in the unsuccessful group (Table 1).

Of the hemodialysis patients, the rates of unsuccessfully reducing the dialysis frequency at 4 and 8 weeks were 33% and 88%, respectively. In the successful group at 4 weeks, 41 (73%) patients failed to maintain a reduction in hemodialysis frequency throughout 8 weeks, and most of them failed at the fifth week. There were some differences observed among the baseline characteristics of patients who were unsuccessful in reducing their hemodialysis frequency over 4 weeks and over 8 weeks, and who were successful in reducing hemodialysis frequency over 8 weeks. However, the results of the Bonferroni multiple-comparison test were not significantly different (Table S1). 

**Figure 1:**
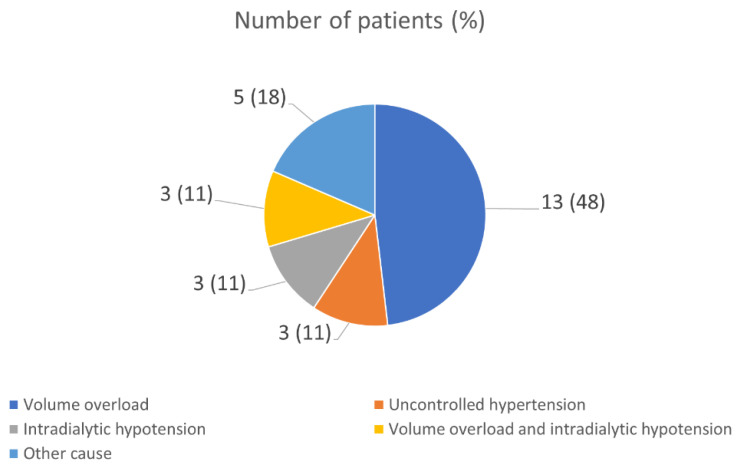
The causes of unsuccessful reductions in hemodialysis frequency. Other causes included uremic symptoms (two patients), alterations of consciousness during dialysis (two patients), and an uncomfortable feeling after the dialysis session (one patient).

The most common cause for an unsuccessful reduction in dialysis frequency at 4 weeks was in volume overload (48.15%) (Figure 1).

### 3.2. Predictors of Unsuccessful Reductions in Hemodialysis Frequency

The univariate logistic regression analysis showed that the predictors of an unsuccessful reduction in dialysis frequency were diabetes mellitus, iPTH level, and pre-dialysis overhydration (i.e., the pre-dialysis body weight minus the dry weight from the BIA) (Table 2).

The multivariate logistic regression analysis demonstrated that the DM (OR 4.37; 95% CI 1.13–16.83; *p*-value = 0.032), CHF (OR 9.71; 95% CI 1.16–81.43; *p*-value = 0.036), pre-dialysis overhydration (OR 2.97; 95% CI 1.23–7.19; *p*-value = 0.016), and dry weight from the BIA (OR 3.41; 95% CI 1.01–11.49; *p*-value = 0.047) were predictors of an unsuccessful reduction in hemodialysis frequency (Table 3). 

### 3.3. Clinical Prediction Score 

The linear equation was log odds (failure reducing hemodialysis frequency) = −3.24 + 1.47 (DM) + 2.27 (CHF) + 1.09 (pre-dialysis overhydration) + 1.23 (dry weight from BIA) − 1.15 (set dry weight) − 0.70 (pre-dialysis body weight) + 0.63 (post-dialysis body weight). The Hosmer–Lemeshow test was performed to test the goodness of fit, which demonstrated a *p*-value of 0.54. The E:O ratio and the AUROC of the developed model were 1.000 and 0.798 (95% CI 0.704–0.893), respectively (Table 4).

For the purposes of internal validation, bootstrapping was performed with a 500-bootstrap sample. The coefficients from the developed model were multiplied by the shrinkage factors of 0.65 (optimism adjusted) (Table 4). The optimism-adjusted linear equation was log odds (failure reducing hemodialysis frequency) = −2.3 + 0.95 (DM) + 1.47 (CHF) + 0.71 (pre-dialysis overhydration) + 0.80 (dry weight from BIA) − 0.75 (set dry weight) − 0.46 (pre-dialysis body weight) + 0.41 (post-dialysis body weight). For the calibration, the E:O ratio and the AUROC of the optimism-adjusted model were 0.997 and 0.728 (95% CI 0.637–0.828), respectively (Table 4).

**Figure 2:**
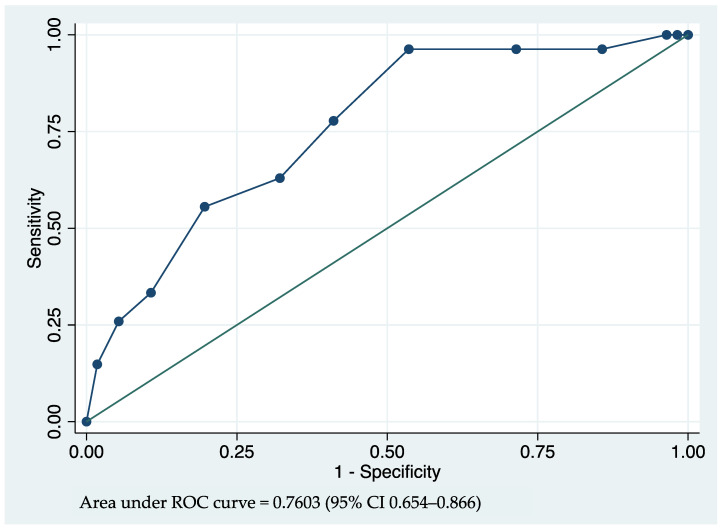
The receiver operating characteristic (ROC) curves and the area under the ROC (AUROC) of the final prediction model for unsuccessful reductions in hemodialysis frequency.

The lowest coefficient of 0.45 was used as a denominator for the other predictors’ coefficients. The results were rounded to integers and used for predicting the score. The weighting scores were assigned as 2 points for a patient with DM, 3 points for a patient with CHF, 2 points per pre-dialysis overhydration in a liter, 2 points per dry weight difference (dry weight from BIA – dry weight in actual dialysis setting) in kilograms, and −1 point for a net ultrafiltration in a liter (post-dialysis body weight − pre-dialysis body weight). The AUROC of the prediction score was 0.760 (95% CI 0.654–0.866) (Table 5, Figure 2).

**Figure 3:**
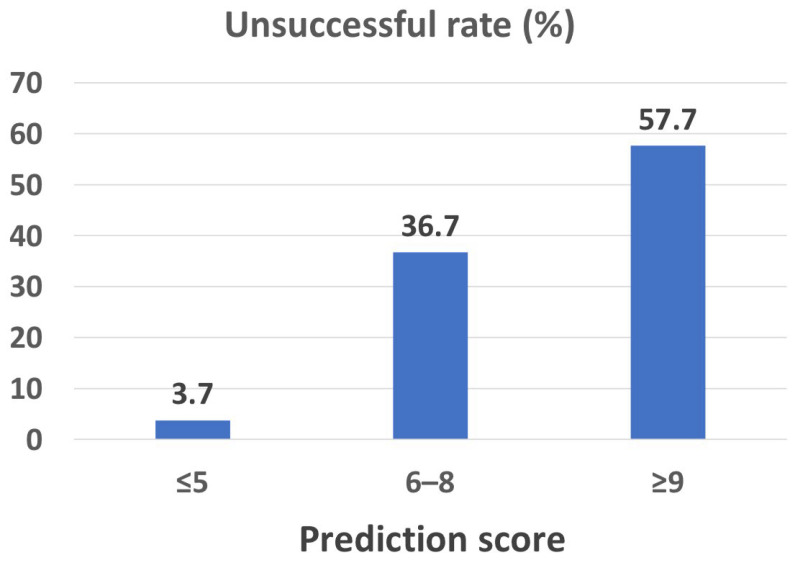
The rate of unsuccessful reductions in hemodialysis frequency at 4 weeks according to the score from the final prediction model.

The score from the final model of 5 or less, 6–8, and 9 or more demonstrated an unsuccessful rate of 3.7%, 36.7%, and 57.7%, respectively (Figure 3). 

## 4. Discussion

This study showed the predictors of unsuccessful reductions in hemodialysis frequency from thrice to twice weekly at 4 weeks during the COVID-19 pandemic, which included DM, CHF, pre-dialysis overhydration, and dry weights that were calculated by BIA. The developed, validated, and final model using these predictors showed a good performance for predicting non-success in terms of reducing hemodialysis frequency.

This study showed that the prevalence of success in reducing dialysis frequency at 4 weeks was about two-thirds. However, only 18% of these patients could achieve this over 8 weeks. Therefore, reducing hemodialysis frequency could theoretically reduce COVID-19 infection transmission for both patients and dialysis staff [6]; however, a reduction in hemodialysis frequency of more than 4 weeks is usually unfeasible. 

In one study, Lodge MDS [10] demonstrated that safe detection could be achieved by temporarily reducing hemodialysis frequency in a pandemic setting. Thrice-weekly hemodialysis patients converted to twice weekly for 4 weeks with no definitive inclusion criteria; suitability was determined by the attending nephrologists. They showed that 68% of patients were able to continue twice-weekly dialysis for a 4-week period. This percentage of successfully reducing hemodialysis frequency was comparable to our study. A retrospective survey of the clinicians suggests that temporarily reducing hemodialysis was preferred for patients with a greater age, lower ultrafiltration requirement, higher residual renal function, pre-dialysis potassium and/or phosphate levels within the normal range, and in patients who were willing to decrease the dialysis frequency. However, the potassium and phosphate levels in our study were not significantly different between the two groups.

The predictors of unsuccessful reductions in hemodialysis frequency in this study can be categorized into non-modifiable factors—which include diabetes mellitus and congestive heart failure—and modifiable factors, which are mostly associated with patients’ hydration status. Therefore, we would suggest that reducing hemodialysis frequency should be cautiously performed in patients who have any of these two comorbidities, especially congestive heart failure and/or previous requirement of high ultrafiltration volume. Moreover, this study also showed that hypervolemic-associated conditions, e.g., volume overload and uncontrolled hypertension, were major causes (81.5%) of failure in reducing dialysis frequency. Thus, a strict control of sodium and water intake should be advised for patients who are in a period of reducing dialysis sessions.

This is the first study to use predictor factors to develop a prediction model of unsuccessful reduction of hemodialysis frequency in a pandemic. The presented model demonstrated good discrimination. Although the COVID-19 infection rate has gradually subsided, this model is still beneficial in other situations where dialysis availability is limited, for example, during a natural disaster, war, or another pandemic. In the aforementioned circumstances, this model can guide clinicians in selecting hemodialysis patients who may encounter fewer complications from hemodialysis frequency reduction. 

There were some limitations in this study. First, this study was a single-center study; therefore, the results might not be representative for other populations, and the predicting risk score might have a limit in terms of its generalizability. Second, the residual urine output, which is an essential factor for volume control, is not available, and this factor might affect the predictability of the model. However, the median dialysis vintage was quite long (4.5 years), and the residual urine output in these patients would likely be small, having a minor effect on the model. Finally, the dry weight being measured by BIA might not be widely available in every dialysis unit. Using other methods to determine the dry weight may not be applicable with respect to this predictive score.

## 5. Conclusions

The prediction score using diabetes mellitus, congestive heart failure, pre-dialysis overhydration, dry weight difference, and net ultrafiltration demonstrated a good performance in predicting unsuccessful hemodialysis frequency reduction at 4 weeks. Our risk prediction score may support physicians’ decisions in choosing a patient who is eligible for hemodialysis frequency reduction.

## Figures and Tables

**Table 1:** Baseline characteristics of the hemodialysis patients.

Characteristics	Successful (*n* = 56)	Non-Successful (*n* = 27)	Total (*n* = 83)	*p*-Value
Age, year, median (IQR)	70.65 (64.0–81.2)	68.3 (60.3–78.6)	69.6 (63.1–80.4)	0.24
Female, *n* (%)	30 (53.6)	14 (51.2)	44 (53.0)	1.00
Vascular access, *n* (%)				0.86
Fistula	36 (64.3)	18 (66.7)	54 (65.1)	
Graft	5 (8.9)	1 (3.7)	6 (7.2)	
Permanent catheter	15 (26.8)	8 (29.6)	23 (27.7)	
comorbidity, *n* (%)				
Diabetes mellitus	27 (48.2)	21 (77.8)	48 (57.8)	0.02
Hypertension	55 (98.2)	25 (92.6)	80 (96.4)	0.25
Dyslipidemia	35 (62.5)	20 (74.1)	55 (66.3)	0.33
Congestive heart failure	2 (3.6)	4 (14.8)	6 (7.2)	0.08
Ischemic heart disease	14 (25.0)	10 (37.0)	24 (28.9)	0.31
Cerebrovascular disease	10 (17.9)	6 (22.2)	16 (19.3)	0.77
Dialysis vintage, year, median (IQR)	4.7 (2.5–7.4)	4.5 (3.0–7.9)	4.5 (2.8–7.5)	0.83
Laboratory, median (IQR)				
Hemoglobin, g/dL	10.9 (10.1–11.6)	10.4 (9.8–11.1)	10.8 (10–11.6)	0.22
White blood cell ^a^, 10^3^/μL	5.7 (5.0–6.9)	5.8 (5.1–7.3)	5.7 (5.0–6.9)	0.70
Platelet, 10^3^/μL	193 (158–227)	197 (131–236)	193 (147–232)	0.83
Sodium ^b^, mmol/L	137 (135–139)	136 (134–139)	137 (134–139)	0.69
Potassium ^b^, mmol/L	4.1 (3.7–4.7)	4.1 (3.8–4.5)	4.1 (3.7–4.5)	0.85
Chloride ^b^, mmol/L	98 (97–100)	98 (96–100)	98 (97–100)	0.89
Bicarbonate ^b^, mmol/L	25 (24–27)	24 (23–25)	25 (23–26)	0.049
Calcium ^c^, mg/dL	9.1 (8.3–9.7)	8.8 (8.1–9.2)	8.9 (8.3–9.5)	0.14
Phosphate ^c^, mg/dL	3.8 (3.2–4.9)	4.3 (3.5–6.1)	3.9 (3.3–5.1)	0.13
iPTH ^d^, pg/mL	582 (385–805)	442 (322–537)	536 (348–735)	0.04
Albumin ^e^, g/dL	3.7 (3.4–3.9)	3.6 (3.45–3.8)	3.6 (3.4–3.9)	0.63
Dialysis adequacy, median (IQR)				
spKt/V	1.91 (1.66–2.09)	1.85 (1.67–2.04)	1.88 (1.67–2.07)	0.52
URR (%)	80.5 (75.7–83.1)	77.8 (75.4–83.3)	80.0 (75.4–83.3)	0.32
nPCR (g/kg/d)	0.99 (0.87–1.26)	1.08 (0.98–1.17)	1.02 (0.88–1.21)	0.32
eqKt/V	1.67 (1.45–1.82)	1.61 (1.46–1.76)	1.65 (1.45–1.81)	0.55
stdKt/V	2.83 (2.42–3.27)	2.9 (2.47–3.13)	2.84 (2.42–3.17)	0.88
Dry weight, kg, median (IQR)				
Dry weight from BIA	56.2 (49.0–65.1)	60.7 (52.7–73.8)	58.2 (50.3–68.7)	0.06
Set dry weight	57.3 (48.5–65.3)	61.5 (52.5–73.5)	58.5 (50.0–69.0)	0.06
Pre-dialysis parameter, median (IQR)				
Pre-dialysis body weight, kg	58.8 (50.5–67.1)	63.5 (54.5–75.3)	60.0 (51.2–71.3)	0.054
Pre-dialysis overhydration, L	1.9 (1.0–2.5)	2.3 (1.6–3.1)	2 (1.3–2.6)	0.01
Interdialytic weight gain, %	3.4 (2.5–4.0)	3.1 (2.6–3.9)	3.3 (2.5–4.0)	0.92
SBP, mmHg	138.2 (126.8–152.9)	146.7 (127.7–158.0)	140 (127.3–155.3)	0.28
DBP, mmHg	61.3 (54.7–68.0)	61.3 (49.3–74.7)	61.3 (53.7–69.0)	0.98
Heart rate, bpm	69.0 (64.2–76.8)	74.0 (65.3–79.3)	71.3 (64.7–78.3)	0.24
Post-dialysis parameter, median (IQR)				
Post-dialysis body weight, kg	57.2 (49.0–65.2)	61.4 (53.1–74.1)	58.4 (49.9–69.0)	0.06
Post-dialysis overhydration, L	0.2 ((−0.6)–0.8)	−0.2 ((−0.6)–0.4)	0 ((−0.6)–0.6)	0.27
SBP, mmHg	151.9 (139.9–162.7)	154.7 (145.7–163.0)	153.3 (140.7–162.7)	0.70
DBP, mmHg	67.0 (60.7–73.7)	68.0 (59.7–73.7)	67.0 (60.7–73.7)	0.76
Heart rate, bpm	67.9 (60.7–74.0)	69.3 (59.7–76.0)	68.0 (60.7–74.3)	0.99
Ultrafiltration, L	1.8 (1.4–2.2)	2.0 (1.5–2.4)	1.9 (1.5–2.2)	0.15
Ultrafiltration rate, mL/kg/h	8.1 (6.0–9.8)	7.5 (6.7–9.1)	8.0 (6.4–9.8)	0.85

Abbreviations: BIA, bioelectrical impedance analysis; iPTH, intact parathyroid hormone; spKt/V, single pool Kt/V; URR, urea reduction ratio; nPCR, normalized protein catabolic rate; eqKt/V, equilibrated Kt/V; stdKt/V, standard Kt/V; SBP, systolic blood pressure; DBP, diastolic blood pressure. ^a^ The missing 5 patients in the success group and 5 patients in the unsuccessful group. ^b^ The missing 4 patients in the success group. ^c^ The missing 6 patients in the successful group. ^d^ The missing 25 patients in the success group and 12 patients in the failure group. ^e^ The missing 15 patients in the successful group and 3 patients in the unsuccessful group. Pre-dialysis overhydration = pre-dialysis body weight—DW from the BIA; post-dialysis overhydration = post-dialysis body weight—DW from the BIA.

**Table 2:** The univariate analyses for unsuccessful reductions in hemodialysis frequency.

	Univariate OR (95% C.I.)	*p*-Value
Age (year)	0.98 (0.94–1.02)	0.32
Female	0.93 (0.37–2.34)	0.88
Comorbidity		
Diabetes mellitus	3.76 (1.32–10.72)	0.01
Hypertension	0.23 (0.02–2.62)	0.24
Dyslipidemia	1.71 (0.63–4.74)	0.30
Congestive heart failure	4.70 (0.80–27.46)	0.09
Ischemic heart disease	1.76 (0.66–4.74)	0.26
Cerebrovascular disease	1.31 (0.42–4.09)	0.64
Laboratory data		
Hemoglobin (g/dL)	0.88 (0.63–1.24)	0.47
Sodium ^a^, mmol/L	0.98 (0.85–1.14)	0.84
Potassium ^a^, mmol/L	0.83 (0.41–1.68)	0.61
Bicarbonate ^a^, mmol/L	0.84 (0.69–1.03)	0.09
Calcium ^b^, mg/dL	0.86 (0.53–1.40)	0.55
Phosphate ^b^, mg/dL	1.25 (0.94–1.68)	0.13
iPTH ^c^, pg/mL	1.00 (0.99–1.00)	0.048
Albumin ^d^, g/dL	0.69 (0.12–3.99)	0.68
Dialysis adequacy		
spKt/V	0.54 (0.10–2.81)	0.46
URR, %	0.94 (0.85–1.04)	0.23
nPCR, g/kg/d	1.50 (0.24–9.39)	0.66
stdKt/V	1.25 (0.64–2.47)	0.51
Dialysis vintage, year	0.98 (0.87–1.09)	0.69
Pre-dialysis overhydration, L	1.82 (1.12–2.96)	0.02
Post-dialysis overhydration, L	0.86 (0.52–1.42)	0.55
Dry weight BIA, kg	1.03 (1.00–1.07)	0.07
Set dry weight, kg	1.03 (1.00–1.07)	0.06
Ultrafiltration, L	1.00 (1.00–1.00)	
Ultrafiltration rate, mL/kg/hour	1.00 (0.83–1.20)	
Pre-dialysis parameter		
Body weight, kg	1.03 (1.00–1.07)	0.06
Interdialytic weight gain, %	1.02 (0.68–1.53)	0.94
SBP, mmHg	1.01 (0.99–1.04)	0.37
DBP, mmHg	1.00 (0.96–1.04)	0.92
Heart rate, bpm	1.03 (0.99–1.08)	0.15
Post-dialysis parameter		
Body weight, kg	1.03 (1.00–1.07)	0.06
SBP, mmHg	1.00 (0.97–1.03)	0.89
DBP, mmHg	0.99 (0.96–1.03)	0.76

Abbreviations: BIA, bioelectrical impedance analysis; iPTH, intact parathyroid hormone; spKt/V, single pool Kt/V; URR, urea reduction ratio; nPCR, normalized protein catabolic rate; eqKt/V, equilibrated Kt/V; stdKt/V, standard Kt/V; SBP, systolic blood pressure; DBP, diastolic blood pressure. ^a^ The missing 4 patients in the successful group. ^b^ The missing 6 patients in the successful group. ^c^ The missing 25 patients in the successful group and 12 patients in the unsuccessful group. ^d^ The missing 15 patients in the successful group and 3 patients in the unsuccessful group.

**Table 3:** The multivariate analyses of the risk factors of unsuccessful reduction in hemodialysis frequency.

Predictors	Univariate OR (95% C.I.)	*p*-Value	Multivariate OR (95% C.I.)	*p*-Value
Diabetes mellitus	3.76 (1.32–10.72)	0.01	4.37 (1.13–16.83)	0.03
Congestive heart failure	4.70 (0.80–27.46)	0.09	9.71 (1.16–81.43)	0.04
Pre-dialysis overhydration (L)	1.82 (1.12–2.96)	0.02	2.97 (1.23–7.19)	0.02
Dry weight BIA (kg)	1.03 (1.00–1.07)	0.07	3.41 (1.01–11.49)	0.047
Dry weight (kg)	1.03 (1.00–1.07)	0.06	0.32 (0.06–1.74)	0.19
Pre-dialysis body weight (kg)	1.03 (1.00–1.07)	0.06	0.50 (0.14–1.72)	0.27
Post-dialysis body weight (kg)	1.03 (1.00–1.07)	0.06	1.88 (0.38–9.25)	0.44

Abbreviations: BIA, bioelectrical impedance analysis.

**Table 4:** The multiple correlation coefficient of the risk factors for unsuccessful reductions in hemodialysis frequency.

	Multivariate Coeff. (95% CI) ^a^	*p*-Value	Multivariate Coeff. (95% CI) ^b^	*p*-Value
Diabetes mellitus	1.47 (0.13 to 2.82)	0.03	0.95 (0.08 to 1.83)	0.03
Congestive heart failure	2.27 (0.15 to 4.40)	0.04	1.47 (0.09 to 2.86)	0.03
Pre-dialysis overhydration (L)	1.09 (0.21 to 1.97)	0.02	0.70 (0.13 to 1.28)	0.02
Dry weight BIA (kg)	1.23 (0.01 to 2.44)	0.047	0.80 (0.01 to 1.58)	0.047
Dry weight (kg)	−1.15 (−2.85 to −0.55)	0.19	−0.75 (−1.85 to 0.36)	0.18
Pre-dialysis body weight (kg)	−0.70 (−0.96 to 2.22)	0.27	−0.46 (−1.26 to 0.35)	0.27
Post-dialysis body weight (kg)	0.63 (−0.96 to−2.22)	0.44	0.41 (−0.62 to 1.44)	0.44

^a^ Developed model; ^b^ optimism-adjusted model. Abbreviations: BIA, bioelectrical impedance analysis.

**Table 5:** The final model of the prediction score for unsuccessful reductions in hemodialysis frequency.

Prediction Factors	Point
Diabetes mellitus	2
Congestive heart failure	3
Pre-dialysis overhydration (per L)	2
Dry weight difference (per kg)	2
Net ultrafiltration (per kg)	−1

## Data Availability

Data are available from the corresponding author (PP) upon reasonable request.

## Supplementary Materials

The following supporting information can be downloaded at: https://www.mdpi.com/article/10.3390/jcm12072550/s1, Table S1: Baseline characteristics of the hemodialysis patients compared between patients who were unsuccessful in reducing hemodialysis frequency over 4 weeks, over 8 weeks and who were successful in reducing hemodialysis frequency over 8 weeks.
