# Supplementary material for: Predictors for Unsuccessful Reductions in Hemodialysis Frequency during the Pandemic
Source: J Clin Med. 2023 Mar 28;12(7):2550. doi: 10.3390/jcm12072550 (PMC10095366; doi:10.3390/jcm12072550)
Supplement: Supplementary file 1 [file jcm-12-02550-s001.zip › jcm-2291307-supplementary.pdf]

Table S1. Baseline characteristics of the hemodialysis patients compared between patients who were unsuccessful in reducing hemodialysis frequency over 4 weeks, over 8 weeks and who were successful in reducing hemodialysis frequency over 8 weeks.

Characteristics	Unsuccessful over 4 weeks (n=27)	Unsuccessful over 8 weeks (n=41)	Successful (n=15)	p-value
Age, year, median (IQR)	68.3 (60.3-78.6)	68.1 (63.8-80.3)	76.5 (69.6-82.5)	0.27
Female, n (%)	14 (51.2)	21 (51.2)	9 (60.0)	0.88
Vascular access, n (%)				0.91
- Fistula	18 (66.7)	27 (65.9)	9 (60.0)	
- Graft	1 (3.7)	4 (9.8)	1 (6.7)	
- Permanent catheter	8 (29.6)	10 (24.4)	5 (33.3)	
comorbidity, n (%)				
- Diabetes mellitus	21 (77.8)	20 (48.8)	7 (46.7)	0.04
- Hypertension	25 (92.6)	41 (100.0)	14 (93.3)	0.17
- Dyslipidemia	20 (74.1)	26 (63.4)	9 (60.0)	0.58
- Congestive heart failure	4 (14.8)	2 (4.90)	0	0.26
- Ischemic heart disease	10 (37.0)	12 (29.3)	2 (13.3)	0.29
- Cerebrovascular disease	6 (22.2)	7 (17.1)	3 (20.0)	0.93
- Dialysis vintage, year, median (IQR)	4.5 (3.0-7.9)	5.1 (2.9-7.1)	3.7 (1.9-7.5)	0.08
Laboratory, median (IQR)				
- Hemoglobin, g/dL	10.4 (9.8-11.1)	10.9 (10.2-11.7)	11.0 (10.0-11.4)	0.71
- White blood cell[a], $10^3/\mu L$	5.8 (5.1-7.3)	5.7 (5.1-6.9)	5.9 (4.7-6.6)	0.49
- Platelet, $10^3/\mu L$	197 (131-236)	190 (161-226)	200 (149-227)	0.46
- Sodium[b], mmol/L	136 (134-139)	137 (135-139)	136 (134-139)	0.61
- Potassium[b], mmol/L	4.1 (3.8-4.5)	4.1 (3.7-4.7)	4.0 (3.8-4.4)	0.07
- Chloride[b], mmol/L	98 (96-100)	98 (97-100)	98 (97-102)	0.31
- Bicarbonate[b], mmol/L	24 (23-25)	25 (24-27)	25 (22-26)	0.47
- Calcium[c], mg/dL	8.8 (8.1-9.2)	9.3 (8.3-9.7)	8.8 (8.5-9.1)	0.02*
- Phosphate[c], mg/dL	4.3 (3.5-6.1)	3.8 (3.3-4.9)	3.9 (3.1-4.6)	0.69
- iPTH[d], pg/mL	442 (322-537)	570 (336-786)	627 (497-981)	0.001*
- Albumin[e], g/dL	3.6 (3.5-3.8)	3.7 (3.4-3.9)	3.5 (3.3-3.8)	0.71
Dialysis adequacy, median (IQR)				
- spKt/V	1.85 (1.67-2.04)	1.84 (1.65-2.04)	1.96 (1.72-2.14)	0.62
- URR (%)	77.8 (75.4-83.3)	79.3 (75.4-82.5)	82.0 (76.7-84.0)	0.67
- nPCR (g/kg/d)	1.08 (0.98-1.17)	0.97 (0.86-1.20)	1.08 (0.88-1.29)	0.27
- eqKt/V	1.61 (1.46-1.76)	1.60 (1.44-1.79)	1.69 (1.49-1.91)	0.50
- stdKt/V	2.9 (2.47-3.13)	2.75 (2.24-3.13)	3.10 (2.58-3.48)	0.046*
Dry weight, kg, median (IQR)				
- Dry weight from BIA	60.7 (52.7-73.8)	58.6 (50.4-68.7)	50.3 (45.2-58.3)	1.18
- Set dry weight	61.5 (52.5-73.5)	58.5 (51.0-69.0)	50.0 (45.5-59.0)	0.20
Pre-dialysis parameter, median (IQR)				
- Pre-dialysis body weight, kg	63.5 (54.5-75.3)	60.7 (51.2-71.3)	51.5 (47.0-60.3)	0.23
- Pre-dialysis overhydration, L	2.3 (1.6-3.1)	2.0 (1.3-2.5)	1.2 (0.8-2.1)	0.21
- Interdialytic weight gain, %	3.1 (2.6-3.9)	3.3 (2.5-3.9)	3.4 (2.2-4.0)	0.40
- SBP, mmHg	146.7 (127.7-158.0)	138.7 (128.7-153.7)	133.0 (121.0-151.0)	0.25
- DBP, mmHg	61.3 (49.3-74.7)	61.3 (55.0-67.0)	60.7 (53.3-70.7)	0.57

- Heart rate, bpm	74.0 (65.3-79.3)	69.0 (63.7-77.0)	69.0 (64.7-74.3)	0.006*
Post-dialysis parameter, median (IQR)				
- Post-dialysis body weight, kg	61.4 (53.1-74.1)	58.4 (51.0-69.0)	49.9 (45.6-59.0)	0.20
- Post-dialysis overhydration, L	-0.2 ((-0.6)-0.4)	0.3 ((-0.2)-1)	-0.2 ((-1.0)-0.3)	0.006*
-SBP, mmHg	154.7 (145.7-163.0)	152.0 (142.0-162.7)	149.7 (138.7-166.7)	0.42
-DBP, mmHg	68.0 (59.7-73.7)	68.7 (60.7-72.7)	62.3 (60.7-77.7)	0.92
- Heart rate, bpm	69.3 (59.7-76.0)	68.0 (62.0-74.3)	67.0 (57.3-71.3)	0.64
Ultrafiltration, L	2.0 (1.5-2.4)	1.9 (1.6-2.2)	1.5 (1.3-2.2)	0.63
Ultrafiltration rate, mL/kg/hour	7.5 (6.7-9.1)	8.0 (69.0-9.8)	8.3 (6.0-9.1)	0.07

*Bonferroni multiple-comparison test was not significantly different.

Abbreviations: BIA, bioelectrical impedance analysis; iPTH, intact parathyroid hormone; spKt/V, single pool Kt/V; URR, urea reduction ratio; nPCR, normalized protein catabolic rate; eqKt/V, equilibrated Kt/V; stdKt/V, standard Kt/V; SBP, systolic blood pressure; and DBP, diastolic blood pressure. [a]The missing 5 patients in the successful group and 5 patients in the unsuccessful group. [b]The missing 4 patients in the successful group. [c]The missing 6 patients in the successful group. [d]The missing 25 patients in the successful group and 12 patients in the unsuccessful group. [e]The missing 15 patients in the successful group and 3 patients in the unsuccessful group. Pre-dialysis overhydration = pre-dialysis body weight—the DW from the BIA; post-dialysis overhydration = post-dialysis body weight—the DW from the BIA.
